# Study of B Cell Repertoire in Patients With Anti-N-Methyl-D-Aspartate Receptor Encephalitis

**DOI:** 10.3389/fimmu.2020.01539

**Published:** 2020-07-29

**Authors:** Jingjing Feng, Siyuan Fan, Yinwei Sun, Zhidong Zhang, Haitao Ren, Wenhan Li, Liying Cui, Bin Peng, Xiaotun Ren, Weihua Zhang, Hongzhi Guan, Jing Wang

**Affiliations:** ^1^CAS Key Laboratory of Mental Health, Institute of Psychology, Beijing, China; ^2^Department of Psychology, University of Chinese Academy of Sciences, Beijing, China; ^3^Department of Neurology, Peking Union Medical College Hospital, Chinese Academy of Medical Sciences and Peking Union Medical College, Beijing, China; ^4^Oumeng V Medical Laboratory, Hangzhou, China; ^5^Department of Neurology, Beijing Children's Hospital, National Center for Children's Health, Capital Medical University, Beijing, China

**Keywords:** anti-NMDAR encephalitis, B cell repertoire, single cell, common clone, diagnosis

## Abstract

Anti-N-methyl-D-aspartate receptor (NMDAR) encephalitis is the most common antibody-mediated encephalitis. There are several studies on B cell repertoire of anti-NMDAR encephalitis in Caucasians. Here, the cerebrospinal fluid (CSF) samples of 12 Chinese patients with first-episode anti-NMDAR encephalitis were collected to investigate the B cell receptor (BCR) binding to NMDAR by single cell amplification of BCR and Sanger sequencing. BCR data of healthy persons, and of patients with anti-leucine-rich glioma inactivated 1 (anti-LGI1) encephalitis, multiple sclerosis (MS), and neuromyelitis optica spectrum disorder (NMOSD) from the public databases were used as control. A heavy chain common clone IGHV1-18^*^04,IGHD1-26^*^01/ IGHD2-2^*^03/IGHD2-8^*^01, IGHJ3^*^02_(CDR3) ARVGSKYGFETFDI was found in 11 of 12 enrolled patients but not in the comparison data set. In addition, 4 shared clonotypes were found among these patients, and three of them contained the common clone. This study also revealed that the antibody gene family usage preference between patients and healthy controls were different, while they had similar antibody mutation rate. Our findings may have potential clinical implications for the diagnosis of anti-NMDAR encephalitis.

## Introduction

Anti-N-methyl D-aspartate receptor (NMDAR) encephalitis is a severe multistage neuropsychiatric syndrome and is associated with cerebrospinal fluid (CSF) IgG antibodies against the NR1 subunit of the NMDAR. In 2007, the target antigens were identified to be the NMDARs by Dalmau et al. (1). Since then, numerous patients with anti-NMDAR encephalitis were identified. Anti-NMDAR encephalitis is the most common form of antibody-mediated encephalitis and occurs more frequently than any individual viral cause of encephalitis in young persons (2). In China, it is reported that about 12.9% of unexplained encephalitis cases are autoimmune encephalitis (AE), of which anti-NMDAR encephalitis accounts for 80% (3).

There are two major immunologic triggers of anti-NMDAR encephalitis widely reported: tumors (usually ovarian teratoma) and herpes simplex encephalitis (4). The possible pathogenesis is that the NMDAR expressed in tumor nervous tissue, or on the surface of neurons in the brain, might be released and handed over to the immune system for processing, and a large number of memory B cells and plasma cells are produced in local lymph nodes. After passing through the blood-brain barrier (BBB), memory B cells undergo a series of antigen-driven changes and then differentiate into mature plasma cells, and finally secrete a large number of antibodies (5). These antibodies mediate capping, cross-linking, and internalization of NMDARs, and the density of NMDARs on the surface of neurons is reduced, resulting in the dysfunction of glutamate neurotransmission and the clinical manifestations of these patients (6). Apart from this, it is of concern that unknown immunologic triggers can be found in about 50% of the patients with anti-NMDAR encephalitis, with neither tumor occurrence nor herpes simplex virus (HSV) infection (5). Thus, the study of these patients is expected to contribute to a further elucidation of the pathogenesis of anti-NMDAR encephalitis.

The main binding site of anti-NMDAR IgG is the N368/G369 amino acids at the N-terminal of NR1 subunit (7, 8). In addition to IgG, Dalmau et al. also found 7% of the patients with anti-NMDAR encephalitis had IgA or IgM antibodies, but only IgG antibodies could cause the reduction of NMDAR at synaptic and non-synaptic levels, and thus only IgG antibodies were pathogenic (9). The main classes of pathogenic antibodies against NR1 are IgG1 and IgG3, and they are synthesized intrathecally (10). The positive rate of antibody (IgG) against NR1 subunit is nearly 100% in the CSF samples of patients, but only 71.4–85.6% in serum samples (11, 12). Therefore, the detection of IgG in CSF is important for the clinical diagnosis of anti-NMDAR encephalitis.

Immune repertoire studies, especially single-cell immune repertoire sequencing, have been used to look for potential diagnostic markers and therapeutic targets for diseases (13–15). Single-cell immune repertoire sequencing can provide more detailed and accurate evidences of changes in immune status of diseases. There are several immune repertoire studies of anti-NMDAR encephalitis in recent years. In these studies, through the analysis of the composition and structure of the antibodies against NR1 subunit, as well as the pathogenicity and affinity of the recombinant antibodies, the antibodies against NR1 were proved to be pathogenic antibodies (16–18). So far there is no report of the immune repertoire of anti-NMDAR encephalitis in Chinese. Besides, there are no immune repertoire studies focusing on anti-NMDAR encephalitis patients of whom the immunologic triggers are unknown. Therefore, we conducted a study of Chinese patients with anti-NMDAR encephalitis by single-cell immune repertoire sequencing, and the immunologic triggers of these patients were unknown. We selected B lymphocytes binding to NR1 subunit from CSF, and we amplified the variable region of single-cell B cell receptor (BCR) to analyze the similarities and differences of immune repertoire among patients. The characteristics of B cell immune repertoire we analyzed include clone cluster, gene family usage preference of variable (V), diversity (D) and joining (J) gene segments, amino acid (AA) characteristics in complementary determining region 3 (CDR3), and antibody mutation rate (19). We compared the data of patients with anti-NMDAR encephalitis, anti-leucine-rich glioma inactivated 1 (anti-LGI1) encephalitis (20), multiple sclerosis (MS), neuromyelitis optica spectrum disorder (NMOSD) (21–23), and public healthy controls. We expect our study will help identify potential diagnostic markers and provide clues to explain the pathogenesis of the anti-NMDAR encephalitis.

## Materials and Methods

### Patients

We consecutively collected the CSF samples with lymphocytosis from 12 patients with first-episode anti-NMDAR encephalitis from November 2018 to June 2019. All the patients fulfilled the diagnostic criteria of definite anti-NMDAR encephalitis proposed by Graus et al. (24). The CSF and serum samples of the patients were tested for anti-NMDAR IgG antibody by indirect immunofluorescence (IIF) using EU 90 cells transfected with the NR1 subunit of the NMDAR complex and immobilized on Biochips, which are commercially available (EUROIMMUN, Lübeck, Germany) (25). Ovarian teratoma was identified in one patient, and no specific triggers were found in the other 11 patients. The clinical information of patients was summarized in Table 1.

**Table 1:** Clinical information of patients.

**Patient ID**	**Range of age (years)**	**Abnormal mental behavior**	**Conscious disturbance**	**Seizure**	**Memory deficit**	**mRS of the peak**	**Tumor**	**Immuno-therapy**	**ICU stay (weeks)**	**Mechanical ventilation**	**CSF antibody titer**	**Serum antibody titer**
PA8	1–5	Yes	No	No	No	4	No	IVIG, corticosteroids	0	No	1:32	Negative
PA11	16–20	No	No	Yes	No	2	No	IVIG	0	No	1:32	Negative
PA13	11–15	No	No	Yes	No	3	No	IVIG, corticosteroids, Rituximab	0	No	1:100	Negative
PA20	56–60	Yes	Yes	Yes	Yes	5	No	IVIG, corticosteroids	4	Yes	1:100	Negative
PA21	1–5	Yes	Yes	Yes	Yes	5	No	IVIG	2	No	1:100	Negative
PA22	6–10	Yes	No	Yes	Yes	3	No	IVIG, corticosteroids, Rituximab	0	No	1:320	1:32
PA23	11–15	Yes	Yes	Yes	Yes	5	No	IVIG, corticosteroids,Rituximab	3	No	1:100	1:10
PA24	26–30	Yes	Yes	Yes	Yes	5	Ovarian teratoma	IVIG, corticosteroids, Plasma exchange	4	Yes	1:320	Negative
PA25	36–40	Yes	Yes	Yes	Yes	4	No	IVIG, corticosteroids	0	No	1:100	Negative
PA29	11–15	Yes	Yes	Yes	Yes	5	No	IVIG, corticosteroids	2	No	1:32	Negative
PA30	26–30	Yes	Yes	Yes	Yes	5	No	IVIG, corticosteroids	1	Yes	1:100	1:10
PA31	26–30	Yes	Yes	Yes	Yes	5	No	IVIG, corticosteroids, Plasma exchange	4	Yes	1:100	Negative

*IVIG, intravenous immunogloblin*.

### CSF Single Cell Isolation

Fresh CSF was transported at 4°C and centrifuged at 1,200 rpm for 10 min immediately. The cells were suspended with 500 μl cryopreservation solution (90%FBS +10%DMSO) and stored at −80°C. Recombinant Protein NR1 (OriGene) was labeled by lightning-link ®FITC (Expedeon) through incubation for 3 h in dark conditions. Before flow cytometry, the cells were thawed into 1 ml FBS (4°C) +15 ml 1640 medium (4°C) and centrifuged at 250 g for 10 min. The supernatant was discarded. Then 1 ml Cell Staining Buffer (Biolegend) and fluorescent antibodies were added. All kinds of antibodies (anti-CD20-percp/Cy5.5, anti-CD27-APC, anti-CD38-PE, Biolegend; NR1-FITC) were added with 1 μl to the cell suspension.

4',6-diamidino-2-phenylindole (DAPI) (3 μmol/L, add 500 μl/tube) staining for about 5 min. Cells were sorted into 8-strip PCR tubes, and each tube contained 4 μl ice-cold cell lysis buffer: 1.86 μl nuclease-free water, 1 μl Oligo dT18 (10 μmol/L), 0.1 μl RNase inhibitor (4U, Applied Biosystems), 0.04 μl Triton X-100 (100 ml/L, Sigma), and 1 μl dNTPmix (10 mm). Using BD FACSAria IIIu flow cytometry, target cells (CD20+NR1+) with negative and weak positive DAPI (indicating the cells were still alive) were selected and placed in liquid nitrogen immediately.

### Single B Cell Receptor Sequencing

Single B cell reverse transcription and BCR amplification were performed using Smart-seq2, as previously described (26, 27). Primers were shown in Supplementary Table 1. The PCR tubes containing lysis buffer and cells were heated at 72°C for 3 min and placed on ice for 1 min immediately after cracking. Six μl reverse transcription system was prepared: 2 μl SuperScript II First-Stand Buffer (5×, Invitrogen), 2 μl betaine (5M, Sigma), 0.9 μl MgCl_2_ (100 mM), 0.25 μl DTT (100 mM), 0.1 μl TSO (100 μM, GENEWIZ), 0.25 μl RNAse inhibitor, 0.5 μl SuperScript II Reverse Transcriptase (Invitrogen). Reverse transcription was performed at 42°C for 90 min; 50°C for 2 min, 42°C for 2 min, 10 cycles; 70°C for 15 min; 12°C, ∞. Two-round nested PCR was carried out using the cDNA products as templates. In the first round, 25 μl PCR system was prepared: 12.5 μl KAPA Ready mix (2×, Kapa Biosystems), 0.5 μl IS primers (10 μM), 0.5 μl CIR2 primers (10 μM each), 10 μl reverse transcription products, 1.5 μl nuclease-free water. In the second round, 25 μl PCR system was prepared: 12.5 μl KAPA Ready mix (2×), 1 μl first-round PCR product, 10.5 μl nuclease-free water, 0.5 μl VH primers for heavy chain of BCR (10 μM each), 0.5 μl JH primers (10 μM each); 0.5 μl VL/VK primers for light chain of BCR (10 μM each), 0.5 μl CH1 primers (10 μM each). Amplifications were at 95°C for 3 min; 98°C 20 s, 60°C 20 s, 72°C 45 s, 25 cycles; 72°C for 5 min, 12°C, ∞. For heavy and light chain, we recovered bands of 400 and 450 bp, respectively (Gel DNA Extraction Kit, OMEGA). Ligation and cloning of PCR products were finished by pEASY®-Blunt Cloning Kit (TransGen Biotech), according to the manufacturer's instructions. After screened by blue-white selection, 3 white positive clones of each chain were selected for Sanger sequencing (GENEWIZ).

To acquire the constant region of BCR so as to confirm the Ig class, we improved our methods by replacing primers modified from previous study (16) (Supplementary Table 2). Fifty μl PCR system was prepared: 32 μl nuclease-free water, 10 μl SF Buffer (5×, with 10 mM MgSO_4_), 1 μl dNTP Mix (10 mM each), 2 μl VH primers (10 μM each), 2 μl CH1 primers (10 μM each), 2 μl first-round PCR product, 1 μl Phanta Super-Fidelity DNA Polymerase (Vazyme). Amplifications were at 95°C for 3 min; 95°C 10s, 58°C 30s, 72°C 30s, 35 cycles; 72°C for 10 min, 4°C, ∞.

### Bioinformatic Analysis of BCR Sequencing Data

According to Change-O toolkit (28), sequence alignments were performed by using IgBLAST (29) to analyze the Sanger sequencing data from our patients, as well as the sequencing data of 4 healthy Chinese peripheral B cells downloaded from the Sequence Read Archive (SRA) database. Since the paired-reads of the public healthy control data contained the same entire antigen-binding CDR3 region, we did not assemble them. The B cells germline database from the international ImMunoGeneTics information system (IMGT) was utilized as reference sequence (30). The four antibody consensus frameworks (FR region, including FR1, FR2, FR3, and FR4) and three complementary determining regions (CDR1, CDR2, CDR3) of BCR were divided by *MakeDb.py*. The functional antibody sequences (not containing stop codons and were in-frame) were obtained by *ParseDb.py*, and the sequences were clone clustered using the *Amino acid model of DefineClones.py*. Then R (version 3.5.1) package *Alakazam* was used to analyze antibody gene family usage preference and AA length of CDR3 including V, D, and J gene segments of the BCR heavy chain. *CreateGermlines.py* and R package *Shazam* were used to analyze mutation rate. R package *Alakazam* and *epade* were used to analyze V-J gene combination. The heat map of distribution of common clones among patients were plotted by R package *Pheatmap*. Venn diagrams were drawn on http://bioinformatics.psb.ugent.be/webtools/Venn/.

To test whether the heavy chain common clone was specific for anti-NMDAR encephalitis, we used CDR3 AA sequence of patients with anti-LGI1 encephalitis (20), another 90 sets of BCR sequencing data from non-Chinese healthy population, 334 sets of BCR sequencing data from patients with multiple sclerosis (MS), and public CDR3 sequence of anti-aquaporin-4 (AQP4) (21–23) as control. Anti-LGI1 encephalitis, MS, and NMOSD are also autoimmune diseases of the central nervous system (CNS). In order to avoid potential bias due to different tools, all BCR repertoire data were analyzed by using MiXCR (31). In addition, we searched the heavy chain common clone in NCBI (32) by the Protein-BLAST tool and in cAb-Rep database (33).

### Statistical Analysis

R function *shapiro.test* was used to determine whether the distribution of heavy chain CDR3 region AA length in these patients was normal, and *P* >0.05 was used as the criterion for normal distribution. Statistical analyses were conducted using the Statistical Analysis System (SAS) version 9.4 for comparing the mutation rate between the patients and healthy people. Analysis of variance (ANOVA), Student's *t* test, or the Wilcoxon test (non-normal distributions) were used to analyze continuous variables. A two-tailed *P* < 0.05 was considered statistically significant.

## Results

### Only a Small Number of NR1 Positive B Lymphocytes Were Present in CSF

**Figure 1:**
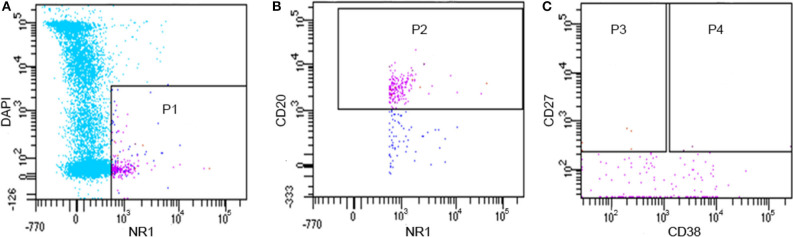
Flow cytometry results of CD20 +NR1+B lymphocytes from one patient (PA11). **(A)** P1: alive NR1 positive cells; **(B)** P2: alive NR1 positive B cells; **(C)** P3: alive NR1 positive memory B cells; **(C)** P4: alive NR1 positive B plasmablast cells.

By flow cytometry, we found that 0.4–1.9% of CSF cells could bind to the NR1 fluorescent antigen, and 0.1–1.4% of B cells in CSF could bind to the NR1 subunit (Figure 1), which was consistent with the result previously reported (16). It should be pointed out that the count of CD20+CD27+CD38-NR1+B memory cells and CD20+CD27+CD38+NR1+ B plasmablast cells were <40 in about 2 ml CSF. Therefore, the majority of the NR1 positive B cells (NR1+CD20+) we obtained from flow cytometry were B cells other than memory B cells and plasmablast cells.

### There Were Common Clones and Shared Clonotypes Presented Among Patients

We finally obtained complete antibody sequences of 83 complete B cells (Supplementary Table 3). For some cells, more than one light chains (with only one heavy chain) or heavy chains (with only one light chain) were obtained, probably because more than one cells were screened by the flow cytometry. We recognized them as complete B cells. For cells having two or more light/heavy chains at the same time, or only having heavy chains or light chains, we recognized them as incomplete B cells, since the types of antibodies cannot be accurately estimated. All incomplete B cells' sequences (Supplementary Table 4) were used for analysis as well.

**Figure 2:**
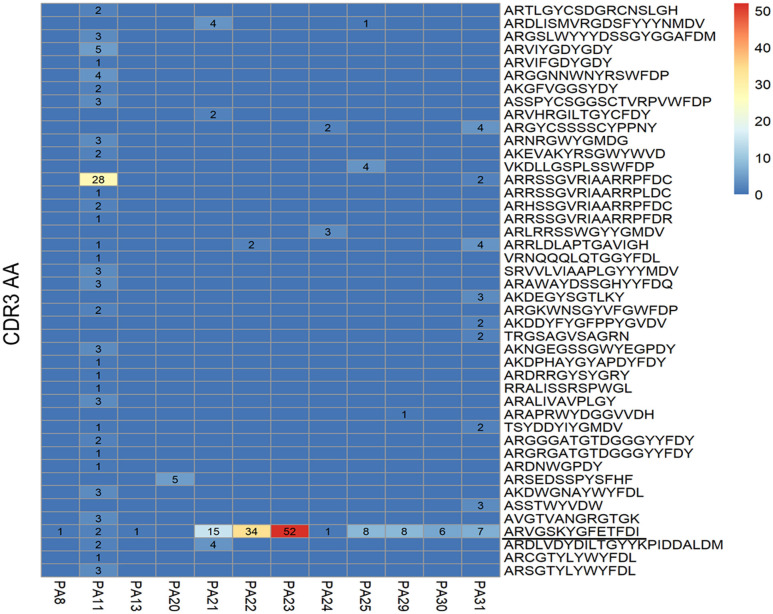
Distribution of heavy chain clones among 12 patients with anti-NMDAR encephalitis. Heat map shows distribution of heavy chain clones among the patients. Numbers > 0 in black represent clone occurrence frequency. PA: patient ID; CDR: complementary determining region; AA: amino acid.

The common clone is defined as the heavy chains' or light chains' V genes and J genes from different cells are the same and the connecting sequence between V genes and J genes translates into the same amino acids. A heavy chain common clone IGHV1-18^*^04,IGHD1-26^*^01/IGHD2-2^*^03/IGHD2-8^*^01,IGHJ3^*^02_(CDR3) ARVGSKYGFETFDI was identified in 11 of 12 patients (Figure 2). The only exception was patient PA20, from whom we only got antibody sequence of one cell. The Ig class of this heavy chain common clone was confirmed as IgG1 in PA21. In addition to the heavy chain clones, we also analyzed the distribution of light chain clones among patients (Supplementary Figure 1).

Four shared clonotypes were found among different patients (Table 2) and three of them contained the heavy chain common clone described above. We adopted the definition of “*clonotype*” as previous described (34), i.e., sequences of B cell recombined variable regions are encoded by the same V genes and J genes of heavy chain, V genes, and J genes of paired light chain, and possessed identical CDR3 AA. Patients PA21, PA22, and PA23 had obvious expansion of the shared clonotype (IGHV1-18^*^04,IGHD1-26^*^01/IGHD2-2^*^03/IGHD2-8^*^01,IGHJ3^*^02_IGLV1-40^*^01/IGLV1-40^*^02,IGJ3^*^02) (Supplementary Table 3). PA11 had clonotype expansion of IGHV4-39^*^01,IGHD6-6^*^01,IGHJ4^*^02_IGLV2-11^*^01,IGJ3^*^02, and that was remarkably different from other patients.

**Table 2:** Shared clonotypes among patients with anti-NMDAR encephalitis.

**Patient ID**	**Heavy chain**					**Light chain**			
	**IGHV**	**IGHD**	**IGHJ**	**CDR3 AA**	**CDR3 _SHM**	**IGLV**	**IGLJ**	**CDR3 AA**	**CDR3_SHM**
PA21 PA22 PA23 PA25 PA29	1-18*04	1-26*01, 2-2*03, 2-8*01	3*02	ARVGSKYGFETFDI	0	1-44*01	3*02	AAWDDSLNGPV	0
PA21 PA22 PA23	1-18*04	1-26*01, 2-2*03, 2-8*01	3*02	ARVGSKYGFETFDI	0	1-40*01, 1-40*02	3*02	QSYDRSLSGYWV	1
PA8 PA22 PA30	1-18*04	1-26*01, 2-2*03, 2-8*01	3*02	ARVGSKYGFETFDI	0	1-44*01	2*01, 3*01	AAWDDSLTGVV	2
PA11 PA31	4-39*01	6-6*01	4*02	ARRSSGVRIAARRPFDC	0	2-11*01	3*02	SSYVRAWV	1

*CDR3 AA, CDR3 amino acids; SHM, Somatic hypermutation*.

### The Most Common Clone of Anti-NMDAR Encephalitis Was Not Found in Healthy People Nor Patients With Anti-LGI1 Encephalitis, MS, or NMOSD

**Figure 3:**
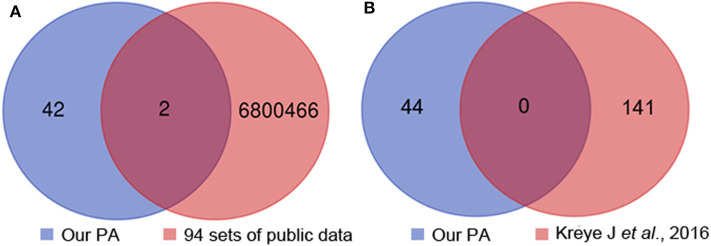
Heavy chain common clones between the 12 patients, 94 sets of healthy people's public data **(A)** and previous report **(B)**. PA, patient.

To provide more evidences the most common clone (IGHV1-18^*^04, IGHD1-26^*^01/IGHD2-2^*^03/IGHD2-8^*^01, IGHJ3^*^02_(CDR3) ARVGSKYGFETFDI) was specifically associated with anti-NMDAR encephalitis, in addition to the 4 sets of healthy Chinese BCR data, we used another 90 sets of non-Chinese healthy people's BCR data from the SRA database. We compared the 44 unique heavy chain clone sequences acquired in 12 patients with the 94 sets of healthy human data and found two heavy chain common clones (CDR3: CARGGNNWNYRSWFDPW, CDR3: CARDNWGPDYW) (Figure 3A), which is not the most common clone (CDR3: ARVGSKYGFETFDI). Meanwhile, the heavy chain sequences acquired in our study were completely different from that reported previously (16) (Figure 3B). Additionally, when we searched the CDR3 AA (ARVGSKYGFETFDI) of the identified common clone for anti-NMDAR encephalitis in NCBI and cAb-Rep database, we didn't find it.

We analyzed 334 sets of BCR sequence data from patients with MS in the SAR database and searched the CDR3 AA (ARVGSKYGFETFDI) of the most common clone. And we also compared our heavy chain common clone with the data of patients with anti-LGI1 encephalitis (20) or NMOSD as reported previously (21–23). However, we didn't find the most common clone in the comparison data set.

### The Antibody Gene Family Usage Preference Was Different Between Patients With Anti-NMDAR Encephalitis and Healthy People

**Figure 4:**
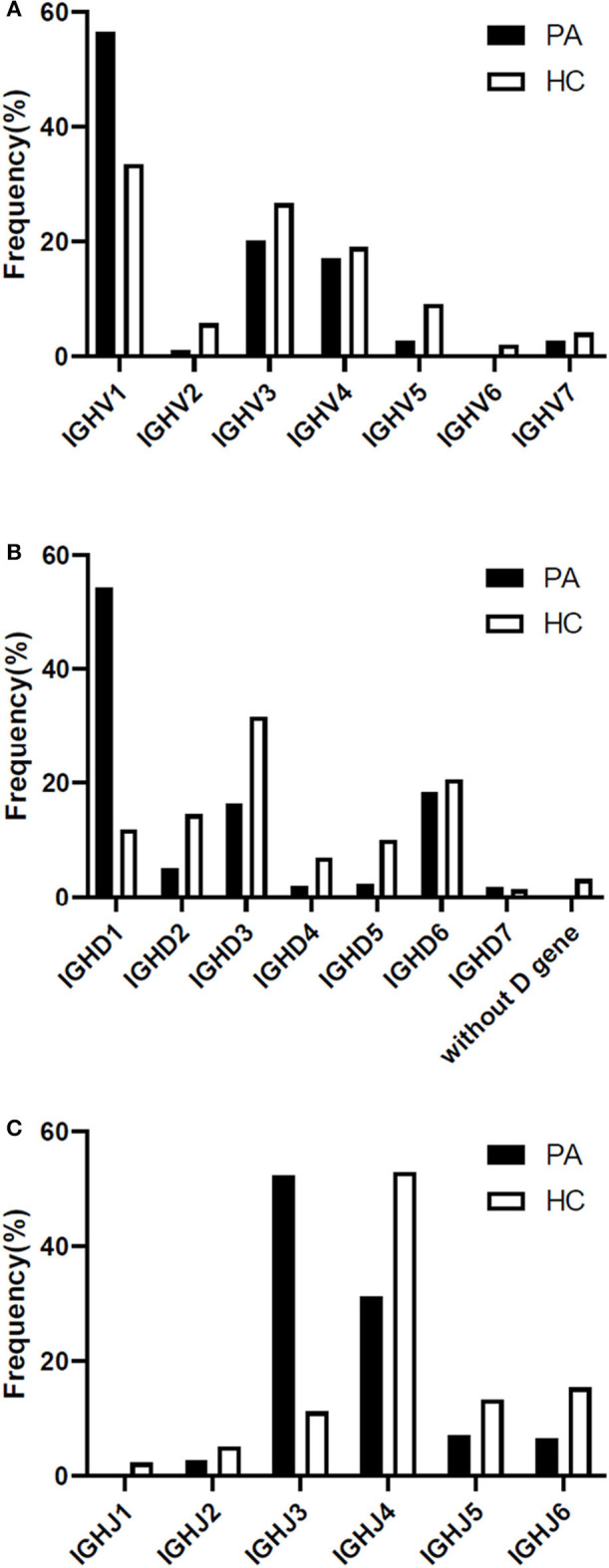
Differences of heavy chain V-D-J gene family usage preference between the patients and 4 healthy Chinese persons **(A-C)**. PA: patient; HC: healthy control; IGH: immunoglobulin heavy chain.

The heavy chain gene usage preferences of patients with anti-NMDAR encephalitis were IGHV1, IGHD1, and IGHJ3 in our study (Figures 4A–C), and antibody gene family IGHV6 and IGHJ1 didn't appear. For light chain, IGLV1 and IGLJ3 were more frequent, and lambda chain was significantly more than kappa chain in these patients (Supplementary Figure 2).

In healthy individuals, the count of the B lymphocytes in CSF is about 0.00 × 10^6^/L−0.03 × 10^6^/L, so we downloaded BCR high throughput sequencing data of all the 4 healthy Chinese from the SRA database as the appropriate control group. Our study had shown that the preference of V-D-J gene family usage in peripheral blood of healthy Chinese were IGHV1/IGHV3, IGHD3, and IGHJ4 (Figures 4A–C).

### Two V-J Gene Combinations Accounted for a Large Proportion in the Heavy Chain of Patients With Anti-NMDAR Encephalitis

**Figure 5:**
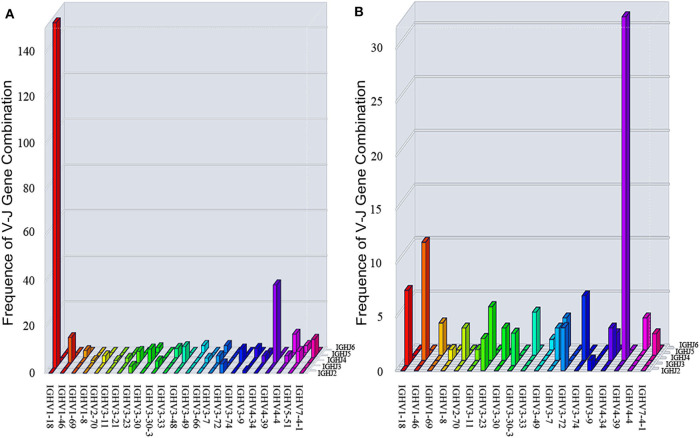
Three-dimensional distribution of V-J gene combination of 12 patients **(A)** and PA11 **(B)**. IGH: immunoglobulin heavy chain.

Two V-J gene combinations accounted for a large proportion in the heavy chain of patients: IGHV1-18^*^04, IGHJ3^*^02; IGHV4-39^*^01, IGHJ4^*^02 (Figure 5A). Because we got a much higher number of B cells from patient PA11 than the other 11 patients, and the most V-J gene combination of heavy chain frequently appeared in PA11 was: IGHV4-39^*^01, IGHJ4^*^02, which was different from that of the other enrolled patients, we showed the V-J gene combination of PA11 separately (Figure 5B).

### There Was No Differences in Mutation Rate of Antibodies Between the Patients With Anti-NMDAR Encephalitis and Healthy People

**Figure 6:**
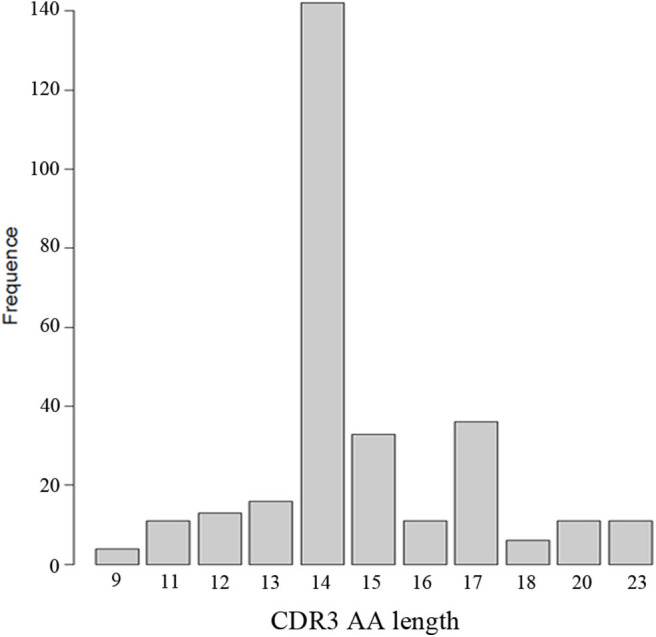
Heavy chain CDR3 amino acid length distribution of the 12 patients.

**Figure 7:**
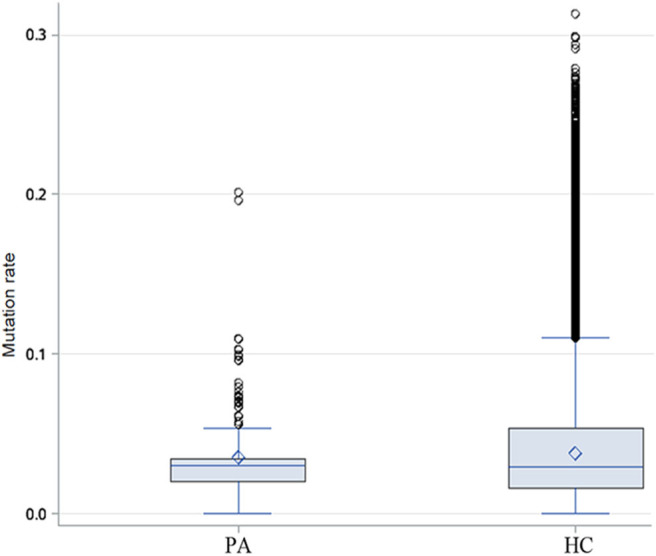
Difference of heavy chain mutation rate between patients and healthy people. Box-and-whisker plots display the minimum value, the first quartile, the median, the third quartile, and the maximum value. PA: patients, HC: healthy control.

Through the analysis of CDR3 AA characteristics of heavy chain, we found that the most popular length was 14, and it accounted for a high proportion, showing skewed distribution by Shapiro-Wilk test (*P* = 1.61 × 10^−16^, Figure 6). The light chain distribution was also not normal. Our study also showed that the total mutation rate of the patient's antibody sequence was low, which was similar to that of healthy people (*P* = 0.4174, Figure 7).

## Discussion

To our knowledge, this is the first study that revealed a heavy chain common clone and the shared clonotypes appeared in most enrolled patients with anti-NMDAR encephalitis. The major findings of our research include: first, a heavy chain common clone of B cell receptor appeared in 11 of 12 patients, but not in healthy people or patients with anti-LGI1 encephalitis, MS, or NMOSD; second, four shared clonotypes presented among different patients with anti-NMDAR encephalitis; third, the V-D-J gene family usage preference of patients with anti-NMDAR encephalitis were different from healthy people, although they have similar antibody mutation rate.

A new strategy was adopted to focus on the NR1 antigen associated B cell repertoire in patients with this encephalitis, that is, pre-sorted B cells bound to NR1 protein by flow cytometry. The number of B cells effectively matched with the heavy and paired light chain was relatively small in our study, due to an indeed small number of B cells go through the BBB of patients, so the immune repertoire of these patients reflected by the results might be the tip of the iceberg. However, we did enrich the B cell repertoire data of the anti-NMDAR encephalitis. Meanwhile, our method had been proved by this study to be effective for studying low-throughput single-cell immune repertoire, which could be applied in diseases like the anti-NMDAR encephalitis.

The most common heavy chain clone IGHV1-18^*^04, IGHD1-26^*^01/IGHD2-2^*^03/ IGHD2-8^*^01,IGHJ3^*^02_(CDR3)ARVGSKYGFETFDI appeared in 11 of 12 patients including PA24 (with ovarian teratoma). This heavy chain common clone was not found in healthy people or patients with anti-LGI1 encephalitis, MS, or NMOSD, suggesting that it is not a common clone for autoimmune diseases of CNS, and it might be a potential diagnostic biomarker for anti-NMDAR encephalitis. Next we will continue to verify whether it presents in anti-NMDAR encephalitis patients with tumor or after herpes simplex virus (HSV) infection, in order to make contributions to the early diagnosis of patients.

Our study revealed four shared clonotypes presented among different patients with anti-NMDAR encephalitis. The shared clonotypes among patients may have the same epitope. For example, PA21, PA22, and PA23 had the shared monoclonal antibody expansion, suggesting that these three boys might had experienced same expansion of selected clonotype, and had similar adaptive immunity response during disease. In contrast, PA11 had expansion of another clonotype, which also appeared in one cell of PA31, showing there might be differences of clonotypes selection during the course of disease among patients. Notably, although PA24 (with ovarian teratoma) did not have a shared clonotype with the other patients, she did have the heavy chain common clone, only with different light chain. In existing literature report (35), heavy chains are sufficient to determine most B cell clonal relationships. So PA24 may have experienced a similar clone selection to the others with unknown triggers that contained the heavy chain common clone.

Previous study had proved that healthy people have diversified antibodies and displayed good Gaussian distribution of heavy chain CDR3 AA length. Antibody gene family usage preference of the four healthy Chinese people in our study was consistent with previous report (36). We revealed the characteristics of the immune repertoire of NR1 positive B cells in patients with anti-NMDAR encephalitis: gene family usage preference was very obvious, which was significantly different from that of healthy people; not all antibody gene families appear, the use of antibody genes was limited, and this was consistent with previous reports (16); and the heavy chain CDR3 AA length of 14 accounted for the largest proportion. All above might be driven by specific antigens. What's more, PA11 had her unique clonotype selection shown by the result of V-J gene combination, and it needs to be verified whether it is related to the different epitopes.

We also proved that most of the anti-NR1 antibodies in CSF of anti-NMDAR encephalitis were hypomutated or non-mutated, and the antibody mutation rate was similar to that of healthy people, which was consistent with the previous report (37), who additionally tested the affinity of monoclonal antibody in CSF of patients and found that low affinity antibodies were the main antibodies in them (38). It is speculated that these hypomutated or non-mutated antibodies belonged to the original sequence in human body, but for some reason, these low affinity antibodies escaped the immune surveillance *in vivo* and could be retained in the body and enter the brain when the BBB was destroyed (37).

This study has limitations. First, patients enrolled are mainly experiencing unknown immunologic triggers. Future studies may also enroll patients with known triggers like tumor or after HSV infection, so as to disclose the disease mechanism from different perspectives. Second, the number of patients in this study is limited. We suggest that future studies can expand the sample size and compare the immune repertoire data of patients with different clinical features (first-episode, recurrent type, and refractory type), so as to reveal how the immune system changes, and to find more meaningful markers for the pathogenesis and prognosis of anti-NMDAR encephalitis.

## Data Availability Statement

Single B cell repertoire sequencing data of patients with anti-NMDAR encephalitis will be provided upon reasonable request. High throughput sequencing data of 4 healthy Chinese peripheral B cells (Accession number: SRX5274837, SRX5274838, SRX5274839, SRX5274840; corresponding sample number: SAMN10786579, SAMN10786580, SAMN10786581, SAMN10786582) and another 90 sets of BCR sequencing data of healthy individuals (SRP188918 and SRP152068) of different populations including Asians and Caucasians were downloaded from the SRA database; 334 sets of BCR sequence data of the MS patients were also downloaded from the SRA database (SRP186647 and SRP042205).

## Ethics Statement

This study was approved by the Institutional Review Board of Peking Union Medical College Hospital (PUMCH) (IRB JS-891). The use of the patients' clinical data and CSF samples was approved by the Ethics Committee of PUMCH. Written informed consent was obtained from each patient or their legal surrogate.

## Author Contributions

JF carried out the single BCR sequencing, data analysis, and drafted the manuscript. SF collected the CSF samples and analyzed the clinical data. YS processed the CSF samples. JF and YS performed the flow cytometry experiments. ZZ supervised the experimental plan. HR, WL, LC, BP, and XR enrolled and diagnosed the patients. HG and JW conceived of and guided the study. All authors participated in the editing and approval of the manuscript.

## Conflict of Interest

The authors declare that the research was conducted in the absence of any commercial or financial relationships that could be construed as a potential conflict of interest.
