# Supplementary material for: Study of B Cell Repertoire in Patients With Anti-N-Methyl-D-Aspartate Receptor Encephalitis
Source: Front Immunol. 2020 Jul 29;11:1539. doi: 10.3389/fimmu.2020.01539 (PMC7403192; doi:10.3389/fimmu.2020.01539)
Supplement: Supplementary file 1 [file Data_Sheet_1.docx]

**Supplementary data**

**Study of B Cell Repertoire in Patients with Anti-N-methyl-D-aspartate Receptor Encephalitis**

Jingjing Feng^1,2^, Siyuan Fan^3^, Yinwei Sun^1,2^, Zhidong Zhang^4^, Haitao Ren^3^, Wenhan Li^4^, Liying Cui^3^, Bin Peng^3^, Xiaotun Ren^5^, Weihua Zhang^5^, Hongzhi Guan^3*^ and Jing Wang^1,2*^

1. CAS Key Laboratory of Mental Health, Institute of Psychology, Beijing, China
2. Department of Psychology, University of Chinese Academy of Sciences, Beijing, China
3. Department of Neurology, Peking Union Medical College Hospital, Chinese Academy of Medical Sciences and Peking Union Medical College, Beijing, China
4. Oumeng V Medical Laboratory, Hangzhou, China
5. Department of Neurology, Beijing Children’s Hospital, Capital Medical University, National Center for Children’s Health, Beijing, China

Correspondence to: J. Wang, PhD

E-mail: wangjing@psych.ac.cn

Correspondence also to: H.Z. Guan, MD

Email: [guanhz@263.net](mailto:guanhz@263.net)

**Supplementary Figures**

**Supplementary Figure 1.** Distribution of light chain clones among 11 patients showing in the heat map (For PA13, only heavy chains were obtained). CDR: complementary determining region; AA: amino acid.


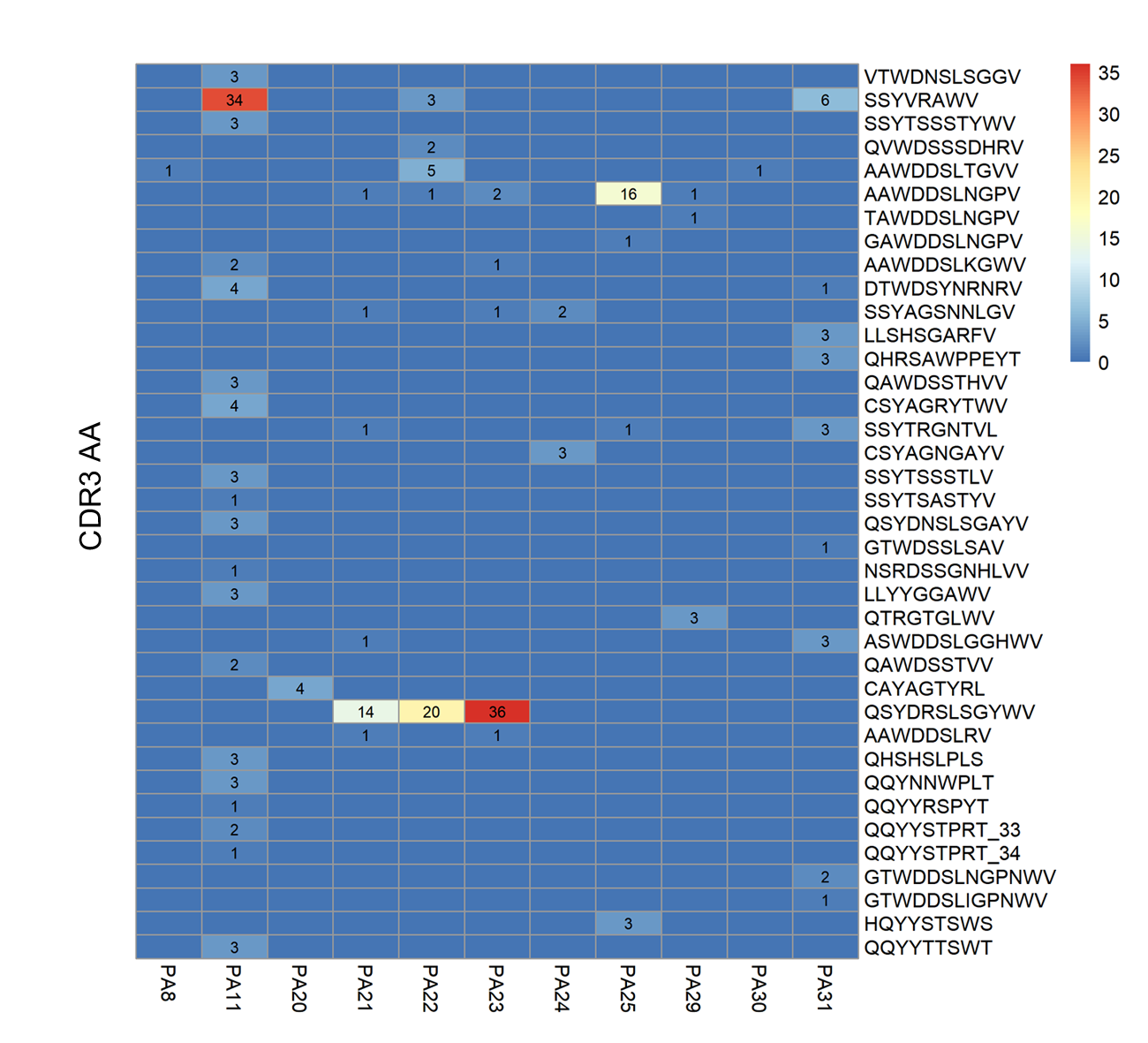


**Supplementary Figure 2.** Light chain V-J gene family usage preference of the patients with anti-NMDAR encephalitis**.** IGL/IGK: immunoglobulin lambda/kappa chain.

**
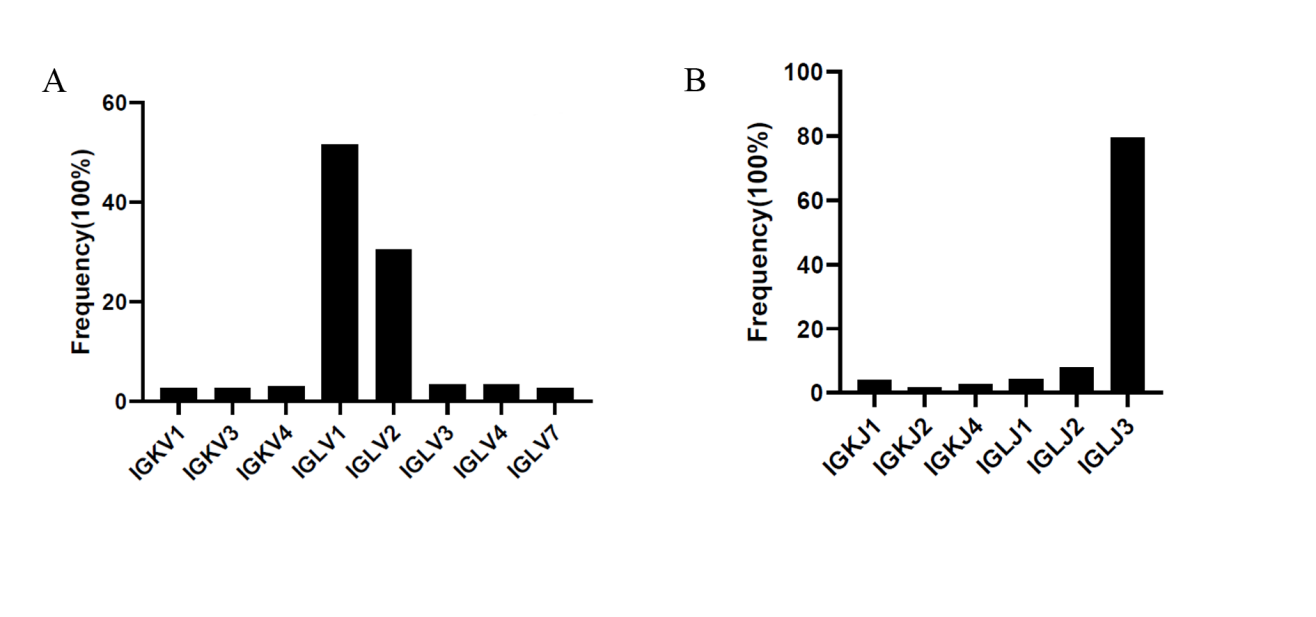
**

**Supplementary Tables**

**Supplementary Table 1.** List of two-rounds PCR primers for single BCR sequencing (a: (rG: Guanine ribonucleotide; +G: LNA-Locked Nucleic Acid)).

| PCR step | Primer Name | Primer Sequence (5’-3’) |
| --- | --- | --- |
| Reverse transcription | TSO | AAGCAGTGGTATCAACGCAGAGTACrGrG +Ga |
|  | oligo-dT18 | TTTTTTTTTTTTTTTTTT |
| 1st PCR | IS | AAGCAGTGGTATCAACGCAGAGTAC |
|  | IGHM-CIR2 | CGGGTACTGCTGATGTCAGAG |
|  | IGHG-CIR2 | TGAGTTCCACGACACCGTCAC |
|  | IGLC-CIR2 | TGTCTTCTCCACGGTGCTCCCT |
|  | IGKC-CIR2 | GGTGACTTCGCAGGCGTAGA |
| Heavy chains 2nd PCR | VH1 | GGCCTCAGTGAAGGTCTCCTGCAAG |
|  | VH2 | GTCTGGTCCTACGCTGGTGAAACCC |
|  | VH3 | CTGGGGGGTCCCTGAGACTCTCCTG |
|  | VH4 | CTTCGGAGACCCTGTCCCTCACCTG |
|  | VH5 | CGGGGAGTCTCTGAAGATCTCCTGT |
|  | VH6 | TCGCAGACCCTCTCACTCACCTGTG |
|  | JH | CTTACCTGAGGAGACGGTGACC |
| Light chains 2nd PCR | VL1/10/3 | TGACTCAGCCACCCTCGG |
|  | VL1-47 | TGACTCAGCCACCCTCAGCGT |
|  | VL1-40/51 | GCAGCCGCCCTCAGTGTCT |
|  | VL2 | CAGTCTGCCCTGACTCAGC |
|  | VL3 | CCCTCGGTGTCAGTGKCCC |
|  | VL4 | CTTGCTGGGAKCCTCGATC |
|  | VL5/9 | AGCCTGTGCTGACTCAGCC |
|  | VL7/8 | CAGACTGTGGTGACYCAGGAGC |
|  | VK1 | ATGACCCAGTCTCCATCTTCCG |
|  | VK102 | CATCCRGATGACCCAGTCTCC |
|  | VK2 | TGATGACCCAGACTCCACTCTC |
|  | VK3/4/5 | TGTTGACRCAGTCTCCAGCCA |
|  | VK6 | TCCAAAGGAGAAAGTCACCATC |
|  | IGLC-CH1 | GAGGAGGGCGGGAACAGAGTG |
|  | IGKC-CH1 | ATGGCGGGAAGATGAAGACAGA |

**Supplementary Table 2.** List of PCR primers for Ig class detection.

| Primer Name | Primer Sequence (5’-3’) |
| --- | --- |
| VH1 | CAGGTGCAGCTGGTGCAG |
| VH1/5 | GAGGTGCAGCTGGTGCAG |
| VH3 | GAGGTGCAGCTGGTGGAG |
| VH3-23 | GAGGTGCAGCTGTTGGAG |
| VH4 | CAGGTGCAGCTGCAGGAG |
| VH4-34 | CAGGTGCAGCTACAGCAGTG |
| Cμ CH1 | GGGAATTCTCACAGGAGACGA |
| IgG CH1 | GTTCGGGGAAGTAGTCCTTGAC |
| Ca CH1 | GTCCGCTTTCGCTCCAGGTCACACT |

**Supplementary Table 3.** Repertoire of human CSF’s complete B cells in anti-NMDAR encephalitis (IGH CDR3 AA: immunoglobulin heavy chain CDR3 amino acids; IGL/IGK CDR3 AA: immunoglobulin lambda/kappa chain CDR3 amino acids).

| Patient ID | Cell ID | Heavy chain | | | | Light chain | | | |
| --- | --- | --- | --- | --- | --- | --- | --- | --- | --- |
|  |  | IGHV | IGHD | IGHJ | IGH CDR3 AA | IGL  /IGK | IGLV  /IGKV | IGLJ  /IGKJ | IGL/IGK CDR3 AA |
| PA8 | Cell1 | 1-18*04 | 1-26*01,  2-2*03,  2-8*01 | 3*02 | ARVGSKYGFETFDI | IGL | 1-44*01 | 2*01,  3*01 | AAWDDSLTGVV |
| PA11 | Cell2 | 4-34*01,  4-34*02 | 5-12*01 | 5*02 | ARGKWNSGYVFGWFDP | IGL | 3-19*01 | 2*01,  3*01 | NSRDSSGNHLVV |
| PA11 | Cell4 | 1-69*13 | 2-15*01 | 5*02 | ASSPYCSGGSCTVRPVWFDP | IGL | 2-14*01 | 1*01 | SSYTSSSTLV |
| PA11 | Cell9 | 3-23*01,  3-23*04,  3-23D*01 | 7-27*01 | 2*01 | AKDWGNAYWYFDL | IGK | 4-1*01 | J2*01 | QQYYRSPYT |
| PA11 | Cell11 | 3-9*01 | 6-13*01 | 2*01 | VRNQQQLQTGGYFDL | IGK | 1-33*01,  1D-33*01 | 4*01 | QHSHSLPLS |
| PA11 | Cell12 | 4-4*02 | 2-15*01 | 6*03 | SRVVLVIAAPLGYYYMDV | IGL | 3-1*01 | 2*01,  3*01 | QAWDSSTHVV |
| PA11 | Cell13 | 3-23*01,  3-23*04,  3-23D*01 | 1-26*01 | 4*02 | AKGFVGGSYDY | IGK | 3-15*01 | 4*01 | QQYNNWPLT |
| PA11 | Cell14 | 3-74*01,  3-74*03 | 7-27*01 | 4*02 | ARDNWGPDY | IGL | 7-43*01 | 3*02 | LLYYGGAWV |
| PA11 | Cell15 | 3-72*01 | 1-26*01 | 2*01 | ARCGTYLYWYFDL | IGK | 1-NL1*01 | 1*01 | QQYYSTPRT |
|  |  | 3-72*01 | 1-26*01 | 2*01 | ARSGTYLYWYFDL |  |  |  |  |
| PA11 | Cell18 | 3-7*01 | 5-12*01,  6-19*01 | 4*01,  4*02,  5*01 | AVGTVANGRGTGK | IGL | 2-11*01 | 3*02 | SSYVRAWV |
| PA11 | Cell19 | 3-7*01 | 6-19*01 | 6*02 | ARNRGWYGMDG | IGL | 2-11*01 | 3*02 | SSYVRAWV |
| PA11 | Cell20 | 1-46*01 | 3-22*01 | 4*02 | ARALIVAVPLGY | IGL | 2-11*01 | 3*02 | SSYVRAWV |
|  |  | 1-46*01,  1-46*03 | 7-27*01 | 4*02 | RRALISSRSPWGL |  |  |  |  |
| PA11 | Cell21 | 4-39*01 | 6-6*01 | 4*02 | ARRSSGVRIAARRPFDC | IGL | 1-51*01 | 3*02 | VTWDNSLSGGV |
| PA11 | Cell22 | 3-74*01,  3-74*03 | 3-22*01 | 4*02 | ARAWAYDSSGHYYFDQ | IGL | 2-11*01 | 3*02 | CSYAGRYTWV |
| PA11 | Cell23 | 1-18*04 | 1-26*01,  2-2*03,  2-8*01 | 3*02 | ARVGSKYGFETFDI | IGK | 4-1*01 | 1*01 | QQYYTTSWT |
|  |  | 4-39*01 | 6-6*01 | 4*02 | ARRSSGVRIAARRPFDC |  |  |  |  |
| PA11 | Cell25 | 4-39*01 | 6-6*01 | 4*02 | ARRSSGVRIAARRPFDC | IGL | 2-11*01 | 3*02 | SSYVRAWV |
| PA11 | Cell26 | 4-39*01 | 6-6*01 | 4*02 | ARRSSGVRIAARRPFDC | IGL | 2-11*01 | 3*02 | SSYVRAWV |
|  |  | 4-39*01 | 6-6*01 | 4*02 | ARRSSGVRIAARRPLDC |  |  |  |  |
| PA11 | Cell27 | 4-39*01 | 6-6*01 | 4*02 | ARRSSGVRIAARRPFDC | IGL | 2-11*01 | 3*02 | SSYVRAWV |
|  |  |  |  |  |  |  | 1-44*01 | 3*02 | AAWDDSLKGWV |
| PA11 | Cell28 | 3-74*01,  3-74*02,  3-74*03 | 2-15*01 | 4*02 | ARTLGYCSDGRCNSLGH | IGL | 2-14*01 | 1*01 | SSYTSASTYV |
|  |  | 7-4-1*02 | 6-13*01 | 5*02 | ARRLDLAPTGAVIGH |  |  |  |  |
| PA11 | Cell29 | 3-23*01,  3-23*04,  3-23D*01 | 6-19*01 | 4*02 | AKNGEGSSGWYEGPDY | IGL | 2-14*01 | 3*02 | SSYTSSSTYWV |
|  |  |  |  |  |  |  | 2-11*01 | 3*02 | SSYVRAWV |
| PA11 | Cell48 | 4-39*01 | 6-6*01 | 4*02 | ARRSSGVRIAARRPFDC | IGL | 2-11*01 | 3*02 | SSYVRAWV |
| PA11 | Cell53 | 4-39*01 | 6-6*01 | 4*02 | ARRSSGVRIAARRPFDC | IGL | 2-11*01 | 3*02 | SSYVRAWV |
| PA11 | Cell55 | 4-39*01 | 6-6*01 | 4*02 | ARRSSGVRIAARRPFDC | IGL | 4-60*03 | 3*02 | DTWDSYNRNRV |
|  |  | 4-39*01 | 6-6*01 | 4*02 | ARRSSGVRIAARRPFDR |  |  |  |  |
| PA11 | Cell56 | 4-39*01 | 6-6*01 | 4*02 | ARRSSGVRIAARRPFDC | IGL | 2-11*01 | 3*02 | SSYVRAWV |
| PA11 | Cell58 | 4-39*01 | 6-6*01 | 4*02 | ARRSSGVRIAARRPFDC | IGL | 2-11*01 | 3*02 | SSYVRAWV |
| PA11 | Cell59 | 3-30-3*01 | 3-22*01 | 3*02 | ARGSLWYYYDSSGYGGAFDM | IGL | 2-11*01 | 3*02 | SSYVRAWV |
|  |  |  |  |  |  |  | 4-60*03 | 3*02 | DTWDSYNRNRV |
| PA11 | Cell60 | 1-46*01 | 4-17*01 | 4*02 | ARVIYGDYGDY | IGL | 2-11*01 | 3*02 | SSYVRAWV |
|  |  |  |  |  |  |  | 3-1*01 | 2*01,  3*01 | QAWDSSTVV |
| PA11 | Cell61 | 1-46*01 | 4-17*01 | 4*02 | ARVIFGDYGDY | IGL | 2-11*01 | 3*02 | SSYVRAWV |
|  |  | 1-46*01 | 4-17*01 | 4*02 | ARVIYGDYGDY |  |  |  |  |
| PA11 | Cell62 | 1-46*01 | 4-17*01 | 4*02 | ARVIYGDYGDY | IGL | 2-11*01 | 3*02 | SSYVRAWV |
|  |  | 4-39*01 | 6-6*01 | 4*02 | ARRSSGVRIAARRPFDC |  |  |  |  |
|  |  | 4-39*01 | 6-6*01 | 4*02 | ARHSSGVRIAARRPFDC |  |  |  |  |
| PA20 | Cell8 | 5-51*01 | 3-22*01 | 4*02 | ARSEDSSPYSFHF | IGL | 2-11*01 | 3*02 | CAYAGTYRL |
| PA21 | Cell2 | 1-18*01 | 3-9*01 | 3*02 | ARDLVDYDILTGYYKPIDDALDM | IGL | 1-40*01,  1-40*02 | 3*02 | QSYDRSLSGYWV |
| PA21 | Cell4 | 1-18*04 | 1-26*01,  2-2*03,  2-8*01 | 3*02 | ARVGSKYGFETFDI | IGL | 1-40*01,  1-40*02 | 3*02 | QSYDRSLSGYWV |
| PA21 | Cell6 | 1-18*04 | 1-26*01,  2-2*03,  2-8*01 | 3*02 | ARVGSKYGFETFDI | IGL | 1-44*01 | 3*02 | AAWDDSLNGPV |
|  |  |  |  |  |  |  | 1-40*01,  1-40*02 | 3*02 | QSYDRSLSGYWV |
| PA21 | Cell9 | 4-4*02 | 3-10*01 | 6*02 | ARDLISMVRGDSFYYYNMDV | IGL | 1-47*01 | 3*02 | AAWDDSLRV |
|  |  |  |  |  |  |  | 2-14*01 | 2*01,  3*01 | SSYTRGNTVL |
| PA21 | Cell11 | 1-18*04 | 1-26*01,  2-2*03,  2-8*01 | 3*02 | ARVGSKYGFETFDI | IGL | 1-40*01,  1-40*02 | 3*02 | QSYDRSLSGYWV |
| PA21 | Cell12 | 4-4*02 | 3-10*01 | 6*02 | ARDLISMVRGDSFYYYNMDV | IGL | 1-40*01,  1-40*02 | 3*02 | QSYDRSLSGYWV |
| PA21 | Cell18 | 3-21*01,  3-21*02 | 3-9*01 | 4*02 | ARVHRGILTGYCFDY | IGL | 1-47*01 | 3*02 | ASWDDSLGGHWV |
|  |  |  |  |  |  |  | 1-40*01,  1-40*02 | 3*02 | QSYDRSLSGYWV |
| PA21 | Cell22 | 1-18*04 | 1-26*01,  2-2*03,  2-8*01 | 3*02 | ARVGSKYGFETFDI | IGL | 1-40*01,  1-40*02 | 3*02 | QSYDRSLSGYWV |
| PA22 | Cell38 | 1-18*04 | 1-26*01,  2-2*03,  2-8*01 | 3*02 | ARVGSKYGFETFDI | IGL | 1-44*01 | 2*01,  3*01 | AAWDDSLTGVV |
| PA22 | Cell40 | 1-18*04 | 1-26*01,  2-2*03,  2-8*01 | 3*02 | ARVGSKYGFETFDI | IGL | 1-40*01,  1-40*02 | 3*02 | QSYDRSLSGYWV |
| PA22 | Cell41 | 1-18*04 | 1-26*01,  2-2*03,  2-8*01 | 3*02 | ARVGSKYGFETFDI | IGL | 1-44*01 | 2*01,  3*01 | AAWDDSLTGVV |
| PA22 | Cell42 | 7-4-1*02 | 6-13*01 | 5*02 | ARRLDLAPTGAVIGH | IGL | 1-40*01,  1-40*02 | 3*02 | QSYDRSLSGYWV |
| PA22 | Cell43 | 1-18*04 | 1-26*01,  2-2*03,  2-8*01 | 3*02 | ARVGSKYGFETFDI | IGL | 1-40*01,  1-40*02 | 3*02 | QSYDRSLSGYWV |
| PA22 | Cell44 | 1-18*04 | 1-26*01,  2-2*03,  2-8*01 | 3*02 | ARVGSKYGFETFDI | IGL | 1-44*01 | 3*02 | AAWDDSLNGPV |
|  |  |  |  |  |  |  | 1-40*01,  1-40*02 | 3*02 | QSYDRSLSGYWV |
| PA22 | Cell45 | 1-18*04 | 1-26*01,  2-2*03,  2-8*01 | 3*02 | ARVGSKYGFETFDI | IGL | 1-40*01,  1-40*02 | 3*02 | QSYDRSLSGYWV |
| PA22 | Cell48 | 1-18*04 | 1-26*01,  2-2*03,  2-8*01 | 3*02 | ARVGSKYGFETFDI | IGL | 1-44*01 | 2*01,  3*01 | AAWDDSLTGVV |
| PA22 | Cell52 | 1-18*04 | 1-26*01,  2-2*03,  2-8*01 | 3*02 | ARVGSKYGFETFDI | IGL | 1-40*01,  1-40*02 | 3*02 | QSYDRSLSGYWV |
| PA22 | Cell78 | 1-18*04 | 1-26*01,  2-2*03,  2-8*01 | 3*02 | ARVGSKYGFETFDI | IGL | 1-40*01,  1-40*02 | 3*02 | QSYDRSLSGYWV |
| PA22 | Cell79 | 1-18*04 | 1-26*01,  2-2*03,  2-8*01 | 3*02 | ARVGSKYGFETFDI | IGL | 2-11*01 | 3*02 | SSYVRAWV |
| PA23 | Cell57 | 1-18*04 | 1-26*01,  2-2*03,  2-8*01 | 3*02 | ARVGSKYGFETFDI | IGL | 1-40*01,  1-40*02 | 3*02 | QSYDRSLSGYWV |
| PA23 | Cell58 | 1-18*04 | 1-26*01,  2-2*03,  2-8*01 | 3*02 | ARVGSKYGFETFDI | IGL | 1-40*01,  1-40*02 | 3*02 | QSYDRSLSGYWV |
| PA23 | Cell59 | 1-18*04 | 1-26*01,  2-2*03,  2-8*01 | 3*02 | ARVGSKYGFETFDI | IGL | 1-40*01,  1-40*02 | 3*02 | QSYDRSLSGYWV |
| PA23 | Cell61 | 1-18*04 | 1-26*01,  2-2*03,  2-8*01 | 3*02 | ARVGSKYGFETFDI | IGL | 1-44*01 | 3*02 | AAWDDSLNGPV |
|  |  |  |  |  |  |  | 1-40*01,  1-40*02 | 3*02 | QSYDRSLSGYWV |
| PA23 | Cell62 | 1-18*04 | 1-26*01,  2-2*03,  2-8*01 | 3*02 | ARVGSKYGFETFDI | IGL | 1-40*01,  1-40*02 | 3*02 | QSYDRSLSGYWV |
| PA23 | Cell63 | 1-18*04 | 1-26*01,  2-2*03,  2-8*01 | 3*02 | ARVGSKYGFETFDI | IGL | 1-40*01,  1-40*02 | 3*02 | QSYDRSLSGYWV |
| PA23 | Cell64 | 1-18*04 | 1-26*01,  2-2*03,  2-8*01 | 3*02 | ARVGSKYGFETFDI | IGL | 1-44*01 | 3*02 | AAWDDSLKGWV |
|  |  |  |  |  |  |  | 1-40*01,  1-40*02 | 3*02 | QSYDRSLSGYWV |
| PA23 | Cell65 | 1-18*04 | 1-26*01,  2-2*03,  2-8*01 | 3*02 | ARVGSKYGFETFDI | IGL | 1-40*01,  1-40*02 | 3*02 | QSYDRSLSGYWV |
| PA23 | Cell67 | 1-18*04 | 1-26*01,  2-2*03,  2-8*01 | 3*02 | ARVGSKYGFETFDI | IGL | 1-40*01,  1-40*02 | 3*02 | QSYDRSLSGYWV |
| PA23 | Cell68 | 1-18*04 | 1-26*01,  2-2*03,  2-8*01 | 3*02 | ARVGSKYGFETFDI | IGL | 1-40*01,  1-40*02 | 3*02 | QSYDRSLSGYWV |
|  |  |  |  |  |  |  | 1-44*01 | 3*02 | AAWDDSLNGPV |
| PA23 | Cell69 | 1-18*04 | 1-26*01,  2-2*03,  2-8*01 | 3*02 | ARVGSKYGFETFDI | IGL | 1-40*01,  1-40*02 | 3*02 | QSYDRSLSGYWV |
| PA23 | Cell70 | 1-18*04 | 1-26*01,  2-2*03,  2-8*01 | 3*02 | ARVGSKYGFETFDI | IGL | 1-40*01,  1-40*02 | 3*02 | QSYDRSLSGYWV |
|  |  |  |  |  |  |  | 2-8*01 | 3*02 | SSYAGSNNLGV |
| PA23 | Cell71 | 1-18*04 | 1-26*01,  2-2*03,  2-8*01 | 3*02 | ARVGSKYGFETFDI | IGL | 1-40*01,  1-40*02 | 3*02 | QSYDRSLSGYWV |
| PA23 | Cell72 | 1-18*04 | 1-26*01,  2-2*03,  2-8*01 | 3*02 | ARVGSKYGFETFDI | IGL | 1-40*01,  1-40*02 | 3*02 | QSYDRSLSGYWV |
| PA23 | Cell73 | 1-18*04 | 1-26*01,  2-2*03,  2-8*01 | 3*02 | ARVGSKYGFETFDI | IGL | 1-47*01 | 3*02 | AAWDDSLRV |
| PA23 | Cell75 | 1-18*04 | 1-26*01,  2-2*03,  2-8*01 | 3*02 | ARVGSKYGFETFDI | IGL | 1-40*01,  1-40*02 | 3*02 | QSYDRSLSGYWV |
| PA23 | Cell76 | 1-18*04 | 1-26*01,  2-2*03,  2-8*01 | 3*02 | ARVGSKYGFETFDI | IGL | 1-40*01,  1-40*02 | 3*02 | QSYDRSLSGYWV |
| PA24 | Cell9 | 5-51*01 | 6-13*01 | 6*02 | ARLRRSSWGYYGMDV | IGL | 2-8*01 | 1*01 | CSYAGNGAYV |
| PA25 | Cell21 | 4-4*02 | 3-10*01 | 6*02 | ARDLISMVRGDSFYYYNMDV | IGL | 1-44*01 | 3*02 | AAWDDSLNGPV |
|  |  | 1-18*04 | 1-26*01,  2-2*03,  2-8*01 | 3*02 | ARVGSKYGFETFDI |  |  |  |  |
| PA25 | Cell27 | 3-9*01 | 3-16*01,  3-16*02,  3-3*01 | 5*02 | VKDLLGSPLSSWFDP | IGL | 1-36*01,  1-44*01 | 3*02 | AAWDDSLNGPV |
|  |  |  |  |  |  |  | 1-36*01,  1-44*01 | 2*01,  3*01,  3*02 | GAWDDSLNGPV |
| PA25 | Cell30 | 1-18*01 | 3-9*01 | 3*02 | ARDLVDYDILTGYYKPIDDALDM | IGK | 4-1*01 | 1*01 | HQYYSTSWS |
|  |  |  |  |  |  | IGL | 2-14*01,  2-14*02,  2-14*03 | 2*01,  3*01 | SSYTRGNTVL |
| PA25 | CellN2 | 1-18*04 | 1-26*01,  2-2*03,  2-8*01 | 3*02 | ARVGSKYGFETFDI | IGL | 1-44*01 | 3*02 | AAWDDSLNGPV |
| PA29 | Cell9 | 1-18*04 | 1-26*01,  2-2*03,  2-8*01 | 3*02 | ARVGSKYGFETFDI | IGL | 4-69*01 | 3*02 | QTRGTGLWV |
| PA29 | Cell18 | 1-18*04 | 1-26*01,  2-2*03,  2-8*01 | 3*02 | ARVGSKYGFETFDI | IGL | 1-44*01 | 3*02 | AAWDDSLNGPV |
|  |  |  |  |  |  |  | 1-44*01 | 3*02 | TAWDDSLNGPV |
| PA30 | Cell40 | 1-18*04 | 1-26*01,  2-2*03,  2-8*01 | 3*02 | ARVGSKYGFETFDI | IGL | 1-44*01 | 2*01,  3*01 | AAWDDSLTGVV |
| PA31 | Cell53 | 3-66*01 | 3-10*01,  3-10*02,  3-16*01 | 4*02 | TRGSAGVSAGRN | IGL | 2-11*01 | 3*02 | SSYVRAWV |
| PA31 | Cell55 | 7-4-1*02 | 6-13*01 | 5*02 | ARRLDLAPTGAVIGH | IGL | 4-60*03 | 3*02 | DTWDSYNRNRV |
| PA31 | Cell60 | 4-4*02 | 3-3*02,  6-13*01,  6-19*01 | 4*02 | ASSTWYVDW | IGL | 7-46*01 | 3*02 | LLSHSGARFV |
| PA31 | Cell61 | 4-39*01 | 6-6*01 | 4*02 | ARRSSGVRIAARRPFDC | IGL | 2-11*01 | 3*02 | SSYVRAWV |
| PA31 | Cell65 | 3-48*03 | 2-2*01,  2-2*02,  2-2*03 | 4*02 | ARGYCSSSSCYPPNY | IGK | 3-11*01 | 2*01 | QHRSAWPPEYT |
| PA31 | Cell66 | 3-49*04 | 3-3*01,  3-9*01 | 6*02 | TSYDDYIYGMDV | IGL | 1-47*01 | 3*02 | GTWDDSLIGPNWV |
|  |  | 7-4-1*02 | 6-13*01 | 5*02 | ARRLDLAPTGAVIGH |  |  |  |  |
| PA31 | Cell70 | 3-30*18,  3-30-5*01 | 3-10*01,  5-18*01,  5-5*01 | 6*02 | AKDDYFYGFPPYGVDV | IGL | 1-47*01 | 3*02 | ASWDDSLGGHWV |
|  |  | 1-18*04 | 1-26*01,  2-2*03,  2-8*01 | 3*02 | ARVGSKYGFETFDI |  |  |  |  |
| PA31 | Cell84 | 3-48*03 | 2-2*01,  2-2*02,  2-2*03 | 4*02 | ARGYCSSSSCYPPNY | IGL | 2-14*01 | 2*01,  3*01 | SSYTRGNTVL |
|  |  | 1-18*04 | 1-26*01,  2-2*03,  2-8*01 | 3*02 | ARVGSKYGFETFDI |  |  |  |  |
| PA31 | Cell86 | 1-18*04 | 1-26*01,  2-2*03,  2-8*01 | 3*02 | ARVGSKYGFETFDI | IGL | 1-51*02 | 3*02 | GTWDSSLSAV |

**Supplementary Table 4.** Repertoire of human CSF’s incomplete B cells in anti-NMDAR encephalitis (IGH CDR3 AA: immunoglobulin heavy chain CDR3 amino acids; IGL/IGK CDR3 AA: immunoglobulin lambda/kappa chain CDR3 amino acids).

| Patient ID | Cell ID | Heavy chain | | | | Light chain | | | |
| --- | --- | --- | --- | --- | --- | --- | --- | --- | --- |
|  |  | IGHV | IGHD | IGHJ | IGH CDR3 AA | IGL  /IGK | IGLV  /IGKV | IGLJ  /IGKJ | IGL/IGK CDR3 AA |
| PA11 | Cell1 | 3-33*01 | 1-7*01 | 5*02 | ARGGNNWNYRSWFDP |  | | | |
| PA11 | Cell5 | 1-18*01 | 3-9*01 | 3*02 | ARDLVDYDILTGYYKPIDDALDM |  | | | |
|  |  | 3-11*01 | 5-18*01,  5-5*01 | 4*02 | ARDRRGYSYGRY |  |  |  |  |
| PA11 | Cell10 | 1-18*04 | 1-26*01,  2-2*03,  2-8*01 | 3*02 | ARVGSKYGFETFDI |  | | | |
|  |  | 3-30*18,  3-30-5*01 | 5-18*01,  5-5*01 | 4*02 | AKDPHAYGYAPDYFDY |  |  |  |  |
| PA11 | Cell24 | 4-39*01 | 6-6*01 | 4*02 | ARRSSGVRIAARRPFDC |  | | | |
|  |  | 3-49*04 | 3-3*01,  3-9*01 | 6*02 | TSYDDYIYGMDV |  |  |  |  |
|  |  |  |  |  |  | IGL | 2-11*01 | 3*02 | CSYAGRYTWV |
|  |  |  |  |  |  |  | 2-11*01 | 3*02 | SSYVRAWV |
| PA11 | Cell30 | 4-34*01,  4-34*02 | 1-1*01,  1-20*01 | 4*02 | ARGGGATGTDGGGYYFDY |  | | | |
|  |  | 4-34*01,  4-34*02 | 1-1*01,  1-20*01 | 4*02 | ARGRGATGTDGGGYYFDY |  |  |  |  |
|  |  |  |  |  |  | IGL | 2-14*01 | 3*02 | SSYTSSSTYWV |
|  |  |  |  |  |  |  | 2-11*01 | 3*02 | SSYVRAWV |
| PA11 | Cell44 | 4-39*01 | 6-6*01 | 4*02 | ARHSSGVRIAARRPFDC |  | | | |
|  |  | 4-39*01 | 6-6*01 | 4*02 | ARRSSGVRIAARRPFDC |  |  |  |  |
| PA11 | Cell46 | 3-30*18,  3-30-5*01 | 6-13*01,  6-19*01 | 4*02 | AKEVAKYRSGWYWVD |  | | | |
|  |  | 4-39*01 | 6-6*01 | 4*02 | ARRSSGVRIAARRPFDC |  |  |  |  |
|  |  |  | | | | IGL | 2-11*01 | 3*02 | SSYVRAWV |
|  |  |  |  |  |  |  | 1-44*01 | 3*02 | AAWDDSLKGWV |
| PA13 | Cell38 | 1-18*04 | 1-26*01,  2-2*03,  2-8*01 | 3*02 | ARVGSKYGFETFDI |  | | | |
| PA24 | Cell10 | 3-48*03 | 2-2*01,  2-2*02,  2-2*03 | 4*02 | ARGYCSSSSCYPPNY |  | | | |
|  |  | 1-18*04 | 1-26*01,  2-2*03,  2-8*01 | 3*02 | ARVGSKYGFETFDI |  |  |  |  |
|  |  |  | | | | IGL | 2-23*01,  2-23*03 | 1*01 | CSYAGNGAYV |
|  |  |  | | | |  | 2-23*01,  2-23*03 | 3*02 | SSYAGSNNLGV |
